# The Anti-Tumor Effects of Adipose Tissue Mesenchymal Stem Cell Transduced with HSV-Tk Gene on U-87-Driven Brain Tumor

**DOI:** 10.1371/journal.pone.0128922

**Published:** 2015-06-12

**Authors:** Suely Maymone de Melo, Simone Bittencourt, Enéas Galdini Ferrazoli, Clivandir Severino da Silva, Flavia Franco da Cunha, Flavia Helena da Silva, Roberta Sessa Stilhano, Priscila Martins Andrade Denapoli, Bianca Ferrarini Zanetti, Priscila Keiko Matsumoto Martin, Leonardo Martins Silva, Adara Aurea dos Santos, Leandra Santos Baptista, Beatriz Monteiro Longo, Sang Won Han

**Affiliations:** 1 Research Center for Gene Therapy (CINTERGEN) of the Universidade Federal de São Paulo, São Paulo, Brazil; 2 Department of Physiology of the Universidade Federal de São Paulo, São Paulo, Brazil; 3 Numpex-Bio, Nucleus of Multidisciplinary Research in Biology of the Universidade Federal do Rio de Janeiro–Xerém, São Paulo, Brazil; 4 Department of Biophysics of the Universidade Federal de São Paulo, São Paulo, Brazil; Rutgers - New Jersey Medical School, UNITED STATES

## Abstract

Glioblastoma (GBM) is an infiltrative tumor that is difficult to eradicate. Treating GBM with mesenchymal stem cells (MSCs) that have been modified with the HSV-Tk suicide gene has brought significant advances mainly because MSCs are chemoattracted to GBM and kill tumor cells via a bystander effect. To use this strategy, abundantly present adipose-tissue-derived mesenchymal stem cells (AT-MSCs) were evaluated for the treatment of GBM in mice. AT-MSCs were prepared using a mechanical protocol to avoid contamination with animal protein and transduced with HSV-Tk via a lentiviral vector. The U-87 glioblastoma cells cultured with AT-MSC-HSV-Tk died in the presence of 25 or 50 μM ganciclovir (GCV). U-87 glioblastoma cells injected into the brains of nude mice generated tumors larger than 3.5 mm^2^ after 4 weeks, but the injection of AT-MSC-HSV-Tk cells one week after the U-87 injection, combined with GCV treatment, drastically reduced tumors to smaller than 0.5 mm^2^. Immunohistochemical analysis of the tumors showed the presence of AT-MSC-HSV-Tk cells only within the tumor and its vicinity, but not in other areas of the brain, showing chemoattraction between them. The abundance of AT-MSCs and the easier to obtain them mechanically are strong advantages when compared to using MSCs from other tissues.

## Introduction

Glioblastoma (GBM) is the most common and lethal primary intracranial tumor. Because GBM is highly invasive and diffusely infiltrates the brain, a complete resection of the tumor is unfeasible. During the last few decades, several alternative therapies have been introduced; however, the mean survival rate remains only 15 months approximately [1]. Suicide genes have been used in clinical gene therapy trials to treat cancers, and the gene encoding the enzyme thymidine kinase from herpes simplex virus-1 (HSV-Tk) is the most commonly used in preclinical and clinical trials against glioma [2–5]. This enzyme has a high affinity to monophosphorylated ganciclovir (GCV), which is further bi- and tri-phosphorylated by endogenous enzymes. During DNA synthesis, the triphosphorylated GCV is incorporated into the DNA strand, blocking chain elongation and leading cells to apoptosis [6, 7]. The HSV-Tk/GCV treatment results in the death not only of the recipient cells (HSV-Tk^+^) but also of surrounding non-recipient tumor cells. This phenomenon is known as the bystander effect and involves the transference of toxic phosphorylated GCV by gap junctions [8–11], apoptotic vesicles [3, 12] and by a paracrine effect that leads to an immunostimulatory response [13–15].

To improve the efficacy of suicide gene therapy, some groups have combined HSV-Tk suicide gene therapy with Temozolomide or radiotherapy, both are commonly used to treat GBM patients. This combination was more effective than HSV-Tk alone [16, 17]. Several studies have used neural stem cells (NSCs) as the recipient of HSV-Tk gene in the treatment of GBM because of their capacity to migrate to the tumor region, even when injected into the contra-lateral hemisphere or in the venous system [18–22]. However, the use of NSC in clinical trials is unfeasible because of the difficulty in obtaining them and of ethical issues surrounding their use.

Mesenchymal stem cells (MSCs) are well-characterized adult stem cells that possess sufficient plasticity to enable them to differentiate into several cell types. Additionally, MSCs can be obtained in large amounts from fat tissue and bone marrow [23–26]. The transplanted MSCs have the ability to migrate to the tumor area and can integrate into tumor vessel walls. This is true even in hypoxic regions resistant to radiotherapy and in tumor infiltrated areas, which typically are difficult to treat [27–35]. These properties make MSCs a good candidate to carry HSV-Tk and to kill GBM. Importantly, the tumoricidal bystander effect of the MSCs modified with HSV-Tk does not harm normal brain tissues surrounding the tumor [36].

Adipose tissue-mesenchymal stem cells (AT-MSC) share common features with MSCs derived from other tissues, such as multipotency in stem cells from mesodermal lineages [37]. The surface marker profile of culture-expanded AT-MSCs resembles the profile of MSCs derived from bone marrow. However, AT-MSCs are distinguished by CD34 expression. Among the important positive aspects of AT-MSCs in comparison to other MSCs are their higher proliferative capacities, the ease in which they can be obtained and their greater abundance [27–28]. These advantages are very significant as we consider the use of these cells in future clinical trials.

Here we present the anti-tumor effects of human AT-MSCs transduced with HSV-Tk genes on the U-87–driven brain tumor model. We used lentivectors for HSV-Tk gene transference because of the high efficiency with which this gene is transduced into stem cells [38, 39]. To the best of our knowledge, this is the first study using AT-MSCs modified with a lentivector carrying HSV-Tk genes, injected at the tumor site, for the treatment of established intracranial glioma.

## Materials and Methods

### Lentivectors’ production and titration

The plasmids used for lentivector production were kindly provided by Professor M.D.V. Laer (Chemotherapeutisches Forschungsinstitut Georg-Speyer-Haus). This work was approved by the Ethics Committee of UNIFESP (under protocol CEP #183974) and UFRJ (AT-MSC cells under the protocol CEP# 145/09). All patients signed the informed consent to participate this study. Plasmid vectors were amplified and purified using Qiagen mega-prep kit (Qiagen,12191). Lentivectors’ production, concentration and titration were performed in a biosafety level 2 (NB2) laboratory, following the protocol established by Naldini et al [38]. Briefly, lentivectors were generated by transfecting human embryonic kidney HEK293T cells by the calcium phosphate method using 20 μg of transfer vector containing Tk-GFP (M488) or only GFP (M107), and 10 μg Gag-Pol (M334), 5 μg Rev, and 6 μg VSV-G envelope (M5). The culture supernatant was collected and filtered after 48 hours. For each 26 mL of supernatant, 4 mL of 20% sucrose solution was added and centrifuged. After centrifugation, the supernatant was discarded and the pellet was resuspended in Dulbecco’s modified Eagle’s medium (DMEM, Sigma, D5030) and stored at -80°C. To determine the viral titer, HEK293T cells were transduced with different concentrations of lentivectors in the presence of 8μg/ml Polybrene (Sigma, 107689). After 3 days, the GFP-positive cells were counted and titers were calculated according to the following formula: Titer (Transduction Units, TU) = % GFP-positive cells x total number of cells on the day of transduction/volume of virus.

### Cell culture, characterization and lentivector transduction

Lipoaspirates were obtained from healthy female donors (n = 3) who underwent abdominal liposuction. The donor ages ranged from 18 to 45 years. The samples were stored at 4°C and were processed to AT-MSCs isolation within 18 hours. AT-MSCs were isolated by the mechanical protocol described previously [40] and plated in tissue culture flasks with α-minimum essential medium (α-MEM, Sigma, 0644) containing 10% fetal bovine serum (FBS; Gibco), 100U/mL penicillin and 100 μg/mL streptomycin (this medium was denominated α-MEMc). Cultures were maintained at 37°C in a humidified atmosphere with 5% CO2 and the medium was replaced every 3 days. At confluence, cells were harvested using a solution of 0.78 mM EDTA and 0.125% trypsin (Gibco, R-001-100) and were re-seeded at a density of 10^4^ cells/cm^2^. Passage was accomplished by dissociation with trypsin followed by reseeding for cell expansion.

Flow cytometry was used to monitor cells for surface marker expression immediately after isolation (fresh samples) and at the first passage. Cell suspensions were incubated with monoclonal antibodies conjugated with fluorescent dyes: CD45–FITC (BD, 341071), CD31–phycoerythrin (PE, BD, 553373), CD146–PE (BD, 562196), CD34-allophycocyanin (APC, BD, 555824), CD34-Peridinin chlorophyll protein (PerCP, BD, 347203), CD105-PE (BD, 562759), CD90-PE (BD, 561970) and CD73-PE (BD, 550741), and with FACS (fluorescence activated cell sorting) lysing solution (BD Biosciences). Flow cytometry analyses were performed using a FACSCalibur (BD Biosciences) and subsequently analyzed using the FACS Diva Software (BD Biosciences).

The adipogenic, osteogenic and chondrogenic potential of AT-MSC preparations were investigated in vitro using the appropriate inducting media, as described previously [40]. Adipogenic differentiation was assessed using Oil Red O staining (Sigma, O0625) and osteogenic differentiation using alizarin red staining (Sigma, A5533). The chondrogenic potential was monitored in pellet cultures. Histological sections of 5 μm were stained with Safranin O (Sigma, S2255) and counterstained with Fast Green (Sigma, F7252) to assess glycosaminoglycan content, as described previously [41].

For lentivector transduction, AT-MSCs passaged 2 or 3 times were plated in a 6-well plate at 1x10^3^ cells/cm^2^ with α-MEMc. Eighteen hours later, the medium was replaced with 1 mL of fresh α-MEMc, and 5–20 μL of a concentrated viral solution (Tk-GFP or GFP) was added in the presence of 8 μg/mL Polybrene. After 24 hours, the medium was replaced with 3 mL of fresh α-MEMc, and the cells were cultured in the incubator for 3 days. The transduction efficiency was determined after this period by counting GFP-positive cells. The transduced cells were stored at -80°C for later use for in vitro and in vivo experiments.

The human primary glioblastoma cell line U-87 was kindly donated by Professor Mari Cleide Sogayar (Department of Biochemistry, Chemistry Institute, Universidade de São Paulo). This cell line was cultured and maintained in DMEM supplemented with 10% FBS, 1% L-glutamine and 1% penicillin.

### In vitro bystander effect assay

U-87 cells and AT-MSC-Tk-GFP or AT-MSC-GFP cells were seeded on a 12 well-plate (1x10^4^ cells of each type per well) with α-MEMc. Every 2 days, the medium was replaced with fresh medium containing 25 μM or 50 μM GCV. After 8 days, cell images were acquired from 5 random fields to determine cell density. The AT-MSC-Tk-GFP cells treated with GCV were photographed again one week later. In the negative control group, GCV was not added to the medium. For non-specific killing activity, U-87 and AT-MSC-GFP cells were cultured separately with GCV. Experiments were performed in triplicate.

### In vivo anti-tumor effect of AT-MSC transduced with HSV-Tk gene on U-87-driven brain tumor

Eight- to 9-week-old nude male mice were purchased from the animal house of the Universidade de São Paulo (São Paulo, SP, Brazil) and maintained in the Central Animal Facility at the Universidade Federal de São Paulo, in accordance with the National Institute of Health Guide for the Care and Use of Laboratory Animals (NIH Publication # 8023, revised in 2011).

GBM tumors were generated in nude mice by injecting 5x10^5^ U-87 cells in 5 μl PBS solution into the right lateral striatum. The animals were anesthetized via the i.p. injection of a ketamine (100 mg/kg) and xylazine (10 mg/kg) solution (Syntec, 1356009 and 1720407) and fastened to a stereotaxic instrument (David Kopf) equipped with a mouse adapter (Enlaup). The microinjection unit was attached to a 10-μl microsyringe (Hamilton) via a water-filled polyethylene tube (PE 10) and the following stereotaxic coordinates were used: 1 mm anterior to the bregma, 1.5 mm lateral to the midline and 3 mm ventral to the skull surface. The infusion was carefully controlled by injecting the solution over 1 μL/min, keeping the microinjection needle in situ for an additional 3 min, suspending 1 mm, waiting 1 more minute (to prevent reflow) and, finally, removing the needle slowly. Seven days after the surgery, the animals were anaesthetized with a ketamine-xylazine solution and divided in 3 groups: Group A (n = 4) was treated with an intratumoral injection of 5μl PBS and, 3 days later, 200 μl PBS were injected in the peritoneum (twice daily, in two rounds of 5 and 3 consecutive days, with 2-day break: control group); Group B (n = 4) was treated with intratumoral injections of 5x10^5^ AT-MSC-HSV-Tk cells diluted in PBS (final volume = 5 μl) and, 3 days later, 200 μl PBS was injected in the peritoneum following the same scheme described above; and Group C (n = 7) was treated with intratumoral injections of 5x10^5^ AT-MSC-HSV-Tk cells and 3 days later 50 mg/kg GCV was injected in the peritoneum following the same scheme as the PBS injection. For the AT-MSC-Tk and PBS intracranial injections, the same coordinates used for U-87 cell injections were used. Soon after the treatments and 12 h later, a drop of ibuprofen (40 mg/ml) was administered orally. These animals were maintained in a climate-controlled room with free access to food and water. Euthanasia was carried out with thionembutal to histology, as described below. All procedures were approved by the Ethics Committee of UNIFESP under the protocol # CEP 83974.

### Histology

One day after the last GCV treatment, the mice were deeply anesthetized with intraperitoneal injection of thionembutal (50 mg/kg) and perfused through the heart with 50 mL of PBS followed by 200 mL of 4% paraformaldehyde at 4°C. The brains were excised and serial coronal brain sections (30 μm thick) were obtained from the whole tumor using a vibratome (Leica, VT1000S) and distributed in a 24-well plate with anti-freezing medium. One slice per well was stained with hematoxylin to identify the slice with the biggest area.

For colocalization of U-87 and AT-MSC, the following antibodies were used for double staining: Human nuclei monoclonal antibody conjugated to FITC to stain both cell types (diluted 1:200, Chemicon International Inc, MAB1281) and anti-GFP antibody conjugated to Alexa Fluor 594 to stain AT-MSCs (diluted 1:500; Molecular Probes, A-21312). Free-floating sections were washed in PBS and permeabilized with 0.5% Triton X-100. Nonspecific binding was blocked with 10% BSA, and followed by incubation with Hu-Nuclei monoclonal antibody diluted in 0.1% BSA containing 0.25% Triton X-100 overnight at 4°C. The next day, the sections were washed with PBS containing 0.25% Tween and incubated with anti-GFP antibody for 2 hours at room temperature. DAPI (Santa Cruz Biotechnology Inc., CA, USA, sc-3598) was used to stain the cell nuclei. The sections were mounted on slides and sealed with coverslips using Fluoromount (Sigma Aldrich, F4680). The sections were analyzed using a confocal scanning laser microscopy (Leica, Nussloch, Germany).

To measure the tumor area, all images were captured using a microscope (20 X magnification, DX53-Olympus America Inc). The slice with the biggest tumor area was chosen, and the tumor area was determined using the delineating tool of Image J software (http://imagej.nih.gov/ij).

### Statistical analysis

Statistical analyses were performed using GraphPad Prism (Version 3.03, GraphPad Software). The data were analyzed using a one-way analysis of variance (ANOVA) followed by Tukey's Multiple Comparison Test. A significance level of 5% was assumed in all comparisons.

## Results

### AT-MSCs culture and HSV-Tk transduction

**Fig 1:**
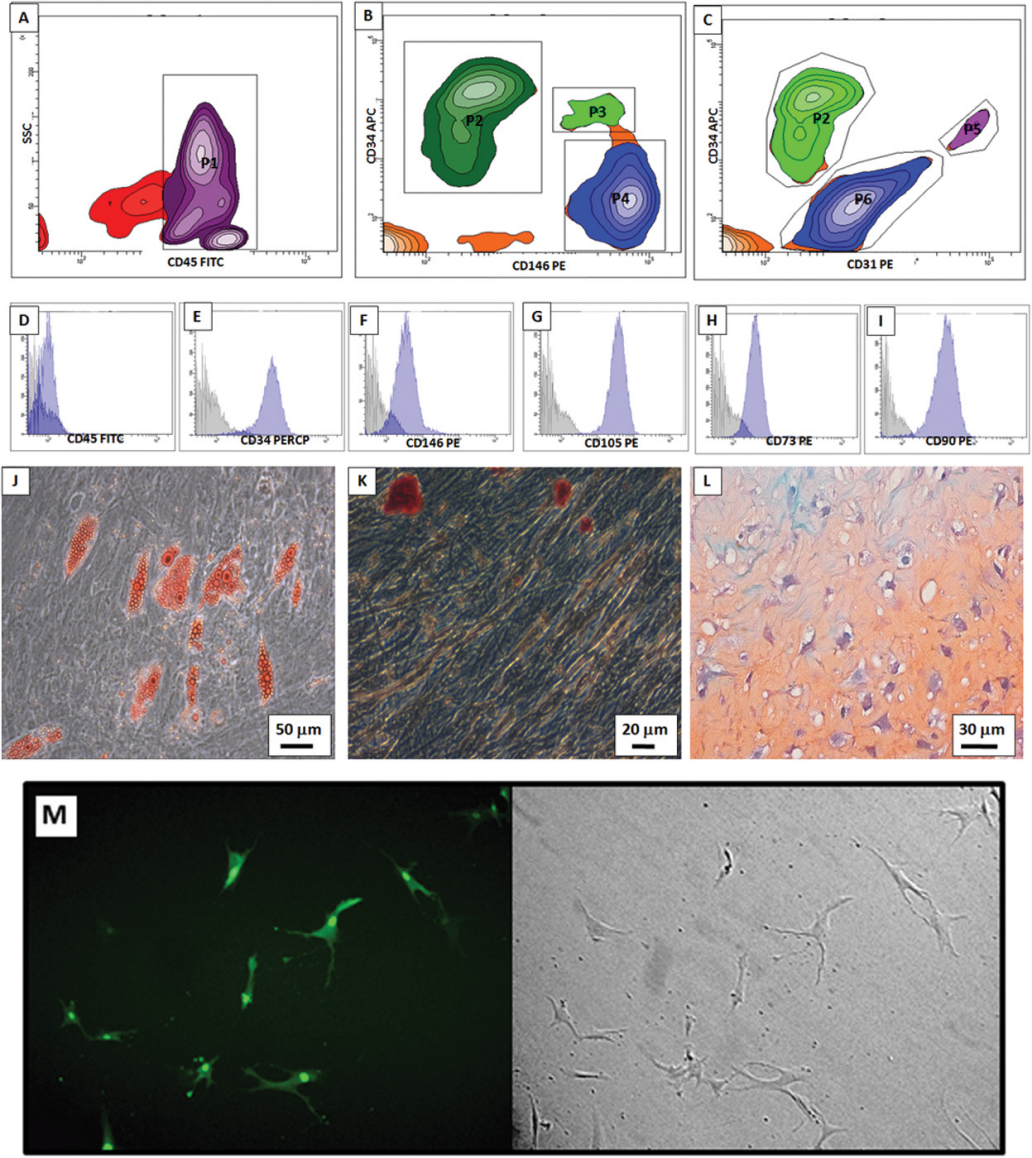
AT-MSC characterizations and Transduction efficiency. In freshly isolated cells major two subpopulations were present: (A) Hematopoietic (P1—CD45 positive) and non-hematopoietic cells (events outside P1, CD45 negative). Non-hematopoietic cells were analyzed in (B) and (C). Pre-adipocytes were detected in B (P2—CD146 negative and CD34 positive) and in C (P2—CD31 negative and CD34 positive). MSC were detected in B (P5—CD146 positive, CD34 negative). Endothelial progenitors were detected in C (P5—CD31 positive, CD34 positive). (D—F) After seeding, AT-MSC from monolayer was still negative for CD45 and positive for CD34 and CD146. (G-I) AT-MSC were also positive for CD105, CD73 and CD90, which are all mesenchymal surface markers in vitro. (J—L) AT-MSC are multipotent for adipogenic (Oil Red O staining), osteogenic (Alizarin staining) and chondrogenic (Pellet culture, safranin staining) lineages, respectively. (M) Transduction efficiency in AT-MSC of the lentivirus containing Tk-GFP is 80%.

AT-MSCs preparations were characterized by flow cytometry and their capacity to differentiate in adipocytes, chondrocytes and osteocytes (Fig 1). In the cells isolated freshly (without culturing), several cell populations were present: non-hematopoietic cells (CD45 negative) (Fig 1A): MSC (Fig 1B), pre-adipocytes (Fig 1B and 1C) and endothelial progenitors (Fig 1C). After culturing, AT-MSCs were still negative for CD45 (Fig 1D). CD34 and CD146 were not expressed homogeneously (Fig 1E and 1F); it is known that the CD34 marker is lost gradually after several passages and the CD146 marker is less expressed in AT-MSC than mesenchymal stem cells from bone marrow [40, 42].

We found a high expression of CD105, CD73 and CD90 (Fig 1G–1I) in the cultured population, which are common MSC markers. Because cell surface markers can be altered depending on their microenvironment, only AT-MSCs preparations passaged three times were used in each assay. Finally, AT-MSCs that were differentiated showed the clear formation of adipocytes, osteocytes and chondrocytes (Fig 1J–1L). The virus titer determined using HEK293T cells was 1.6x10^6^ TU/ml, but this titer was reduced slightly to approximately 80% using AT-MSK-Tk cells as a target (Fig 1M).

### In vitro bystander effect on AT-MSCs and U-87 cells

**Fig 2:**
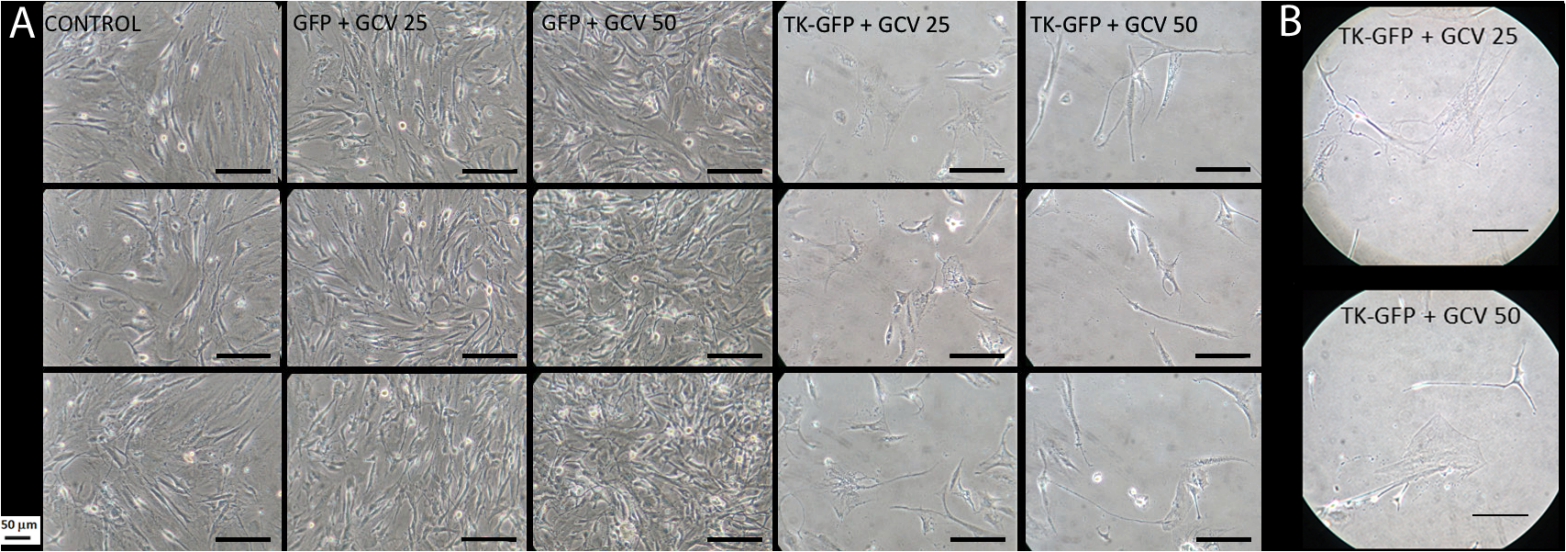
Bystander effect of transduced AT-MSC-Tk in U-87 cells. U-87 cells were co-cultured in each well with the indicated AT-MSC cells at the same proportion. GCV 25 and GCV 50 indicate 25 μM and 50 μM of GCV, respectively; CONTROL indicates co-culture of U-87 and AT-MSC-GFP without GCV; GFP and TK-GFP indicate AT-MSC-GFP and AT-MSC-GFP-Tk cells, respectively. (A) Cell images were acquired, using an inverted microscope 8 days after GCV addition. (B) AT-MSC-Tk + GCV (25 and 50 μM) on the 15th day of culture. Bar = 50 μm.

The culture of U-87 and AT-MSC-GFP cells, together or separately, in the presence of GCV did not affect their growth rate or cell morphology, and cells reached confluence on the 8^th^ day (Fig 2-A). The co-culture of these cells without GCV had a very similar result, showing that GCV did not affect cellular growth. However, the co-culture of U-87 and AT-MSC-GFP-Tk cells in the presence of 25 μM or 50 μM GCV killed most of the cells in one week (Fig 2-A). After 2 weeks, there were still some cells attached to the plate, but most of them changed fibroblast-like morphology to a form with thin and elongated cytoplasm or flattened cytoplasm, which are characteristics of unviable cell morphology (Fig 2-B). To investigate the cellular viability, these cells were maintained in culture for one week further without GCV. During this time, more cells died and no cell growth was observed (not shown). Because lentivector transduction efficiency was approximately 80%, the death of almost all non-transduced AT-MSC-GFP and U-87 cells demonstrates a clear and efficient bystander effect.

### AT-MSC-Tk gene therapy in U-87-driven brain tumors

**Fig 3:**
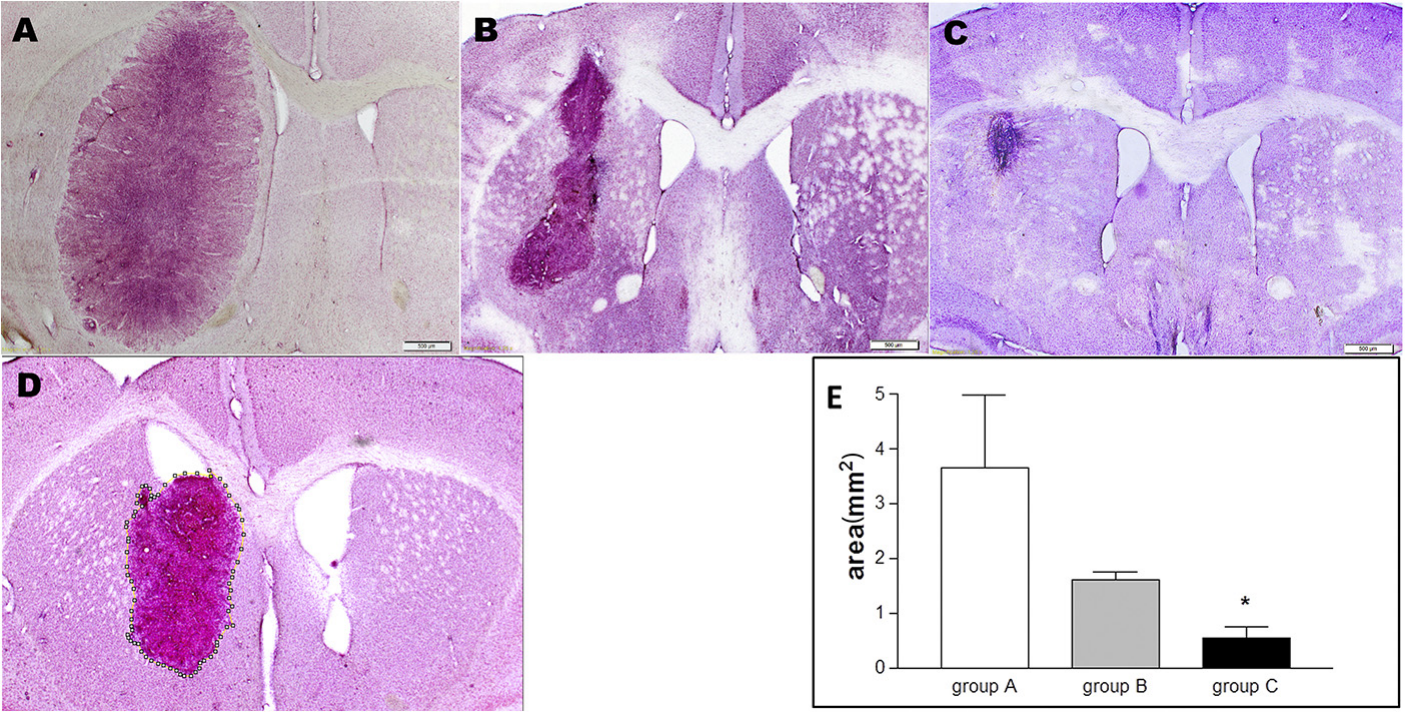
Tumor areas after gene therapy. Ten days after AT-MSC or PBS injection, mice were sacrificed to determine tumor area by histology. Tissue samples were stained with hematoxylin. All mice received U-87 via intracranial injection and treated with:(A): PBS (IP); (B): AT-MSC-Tk (IC) and PBS (IP); (C): AT-MSC-Tk (IC) and GCV (IP). (D): a sample of tumor demarcation using Image J software to determine tumor area. (E) Tumor areas determined from all mice. * p = 0.0136 (group A vs group C). IC: intracranial injection; IP: intraperitoneal injection.

The U-87-driven brain tumors in the animals treated with intracranial injection of AT-MSC-Tk cells followed by the intraperitoneal administration of GCV were very small compared to the tumors from control groups; this difference was significant (Fig 3). Mice treated with AT-MSC-Tk cells without GCV presented a nonsignificant, small reduction of tumor size relative to the PBS-treated group, and these tumors were bigger (median = 1.6 mm^2^) than those of the GCV-treated group (median = 0.5mm^2^). Fig 3E shows the demarcation of a tumor from a slide using Image J software to determine the tumor area.

**Fig 4:**
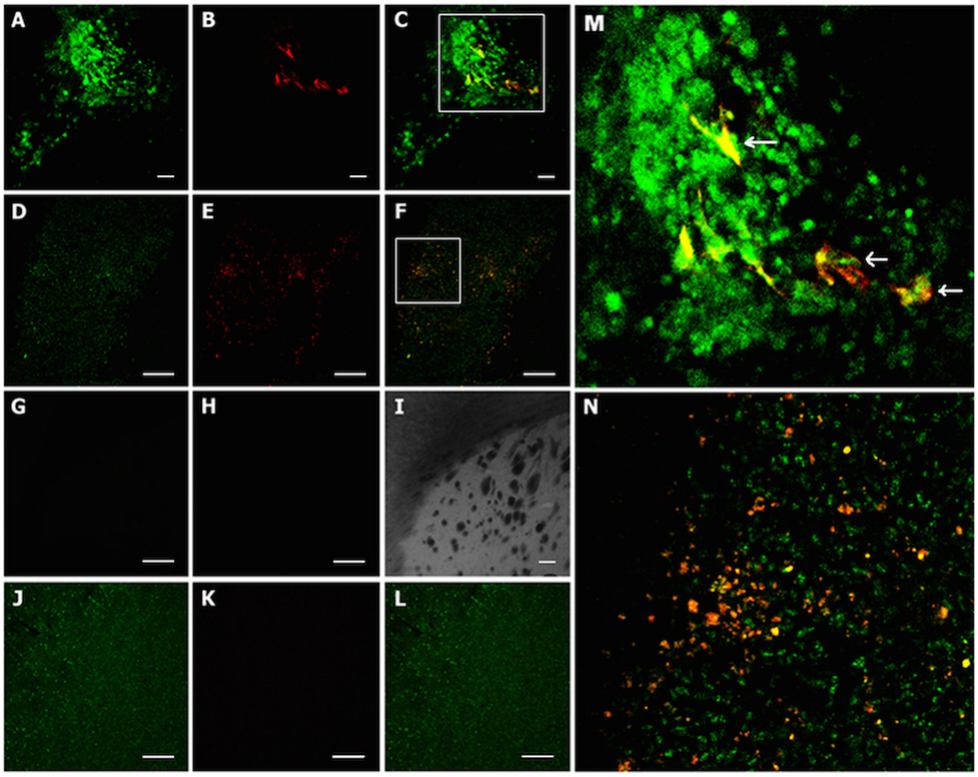
Co-localization of tumor and AT-MSC-Tk by immunohistochemistry. U-87 tumor and AT-MSC were labeled with human nuclei monoclonal antibody conjugated to FITC (green) and AT-MSC-Tk-GFP labeled with anti-GFP antibody conjugated to Alexa Fluor 594 (red). Therefore, AT-MSC double labeled is in yellow. (A-C) U-87 + AT-MSC-Tk + GCV (200 X); (A) anti-Hu; (B) Anti-GFP; (C) Merge; (D-F) U-87 + AT-MSC-Tk no GCV (100 X); (D) anti-Hu; (E) Anti-GFP; (F) Merge;(G-I) Brain slices from the contralateral hemisphere (200 X); (G) anti-Hu; (H) Anti-GFP; (I) Merge of G and I under visible light. (J-L) human brain tissue (100 X); (J) anti-Hu; (K) Anti-GFP; (L) Merge; (M) Digital amplification of the square area of the Fig 4C; (N) Digital amplification of the square area of the Fig 4F. White arrows indicate areas where tumor cells died by treatment, supposedly. Bar = 50 μm.

Brain tissue slices from groups B and C (of the Fig 3) were processed for immunostaining. U-87 and AT-MSC-Tk-GFP were marked in green using human nuclei monoclonal antibody conjugated to fluorescein isothiocyanate (FITC), and to differentiate between these two cell types, anti-GFP antibody conjugated to Alexa Fluor 594 was used to stain the AT-MSC-Tk-GFP cells red. In the amplified image from Fig 4M several round-shaped empty areas (indicated with white arrows) near yellow-labeled cells can be noted. These empty areas are evidence of the dead U-87 cells. As a negative control, slices from the contralateral hemisphere were used. Because staining with both antibodies was negative, we show image Fig 4I using visible light. As a positive control, slices from human brain samples were used. Here, as expected, only human nuclei monoclonal antibodies stained these slices (Fig 4J–4L).

## Discussion

HSV-Tk suicide gene therapy with GCV has been extensively investigated in the treatment of malignant gliomas [8–11]. The use of MSCs as vehicles to carry the HSV-Tk gene is advantageous because MSCs are chemotaxic to tumor cells and they integrate into tumor vessel walls [27–35]. Because MSCs change their own phenotype easily when influenced by their microenvironment, it is very important to validate their identity before using them in experiments by evaluating their specific cell surface markers and differentiation capacities [37]. In this study, surface marker profiles were monitored before (freshly isolated cells) and after cellular adhesion to plastic dishes. Freshly isolated cells and AT-MSCs, obtained by a mechanical protocol, showed all typical surface markers of adipose tissue stem and progenitor cell populations and were capable of differentiating into adipocytes, osteoblasts and chondroblasts confirming their identity as AT-MSCs.

Collagenase is the enzyme most widely used to disrupt lipoaspirate tissue for obtaining AT-MSCs. Partially purified collagenase often contains endotoxin, other peptidases and xenoproteins. In addition, a significant lot variability [43] and the high cost of collagenase for clinical cell therapy are still major technical challenges that have not yet been solved. Mechanical dissociation of the adipose tissue layer from the lipoaspirate samples eliminates exogenous contamination and is a reproducible and inexpensive procedure [44]. Therefore, the use of AT-MSCs as a vehicle for gene transference that were isolated by the mechanical protocol developed by our group [40] is a promising system for ex vivo gene therapies and even for future human clinical trials.

One of main obstacles of transforming MSCs to carry genes is that MSCs and AT-MSCs are refractory to genetic modification. Among available vectors, the lentivectors are considered the most efficient for gene transference and less harmful to target stem cells [45]. Imperceptible cell mortality and the maintenance of phenotype after lentivector transduction indicate low or insignificant side effects of the gene transfer procedure used here. Other concerns regarding the use of MSCs for suicide gene therapy are their sensitivity to GCV and their capacity to permeate phosphorylated GCV to neighboring cells, a phenomenon known as the bystander effect. The elimination of almost all non-modified U-87 and AT-MSC cells after co-culturing with AT-MSC-Tk in the presence of GCV is the best proof of the bystander effect.

In evaluating the therapeutic effect of the AT-MSC-Tk/GCV system in U-87 derived tumors in mice, the group consisting of AT-MSC-Tk cells treated with PBS was included to evaluate the effects of these cells alone in U-87 derived tumors. As was shown in the Fig 3, AT-MSCs alone were not able to significantly reduce tumor size, unlike the group treated with GCV. A similar observation was made by other authors [31, 33]. Although the antitumor effect of MSCs is largely unknown, it was seen that MSCs could inhibit glioma growth when co-injected with tumor cells [29]. However, such analysis must be cautious because the activity of MSCs on tumors is not uniform and can also promote tumorigenesis [35], although this has not observed in established gliomas [33].

In contrast, the tumor reduction by GCV was much more effective than with PBS (Fig 3); however, in our experimental conditions the U-87-derived tumors were not eliminated completely from any of the mice. We posit that because the number of AT-MSC-Tk cells injected is directly related to the efficacy of tumor elimination, it is probable that 5x10^5^ AT-MSC-Tk cells were not sufficient to halt the growth of the same number of U-87 tumor cells. We chose this number of cells because, usually, no more than 5 μl of cell suspension are injected into the adult mice brain so as not seriously disrupt cerebral physiology, and if more cells are added to 5 μl, the solution becomes viscous and cells are lost during and after injection. In addition, even if the same number of both cell types were injected, because AT-MSC-Tk cells were injected a week after U-87 cells, it is likely there were much more U-87 cells in the brain at the moment of injection of AT-MSC-Tk cells. Therefore, it seems that an imbalance of AT-MSC-Tk / U-87 in favor of U-87 was the main reason for incomplete tumor eradication.

Another important point that can explain the lower than expected therapeutic effect is that we used nude mice in our experiments to avoid immune reactions against human MSCs and U-87 cells. Although the use of T-cell deficient mice was necessary here, the cytotoxic bystander effect is partially dependent on T cells [14, 46]. The mechanism of T-cell dependency in enhancing the bystander effect is still not well known.

Although the difference in tumor sizes between the groups treated with AT-MSC-Tk (with and without GCV treatment) is not statistically significant, if we analyze each mouse individually, we note that 5 out of 7 mice in the AT-MSC-Tk treated with GCV group have tumor sizes smaller than 0.5 mm^2^, whereas in the untreated group the smallest area is approximately 1.3 mm^2^, showing a clear difference in the sizes of tumors between these groups.

One of reasons for using MSCs in suicide gene therapy is that MSCs are chemoattracted to tumor cells [26, 29, 32, 33, 47, 48, 49]. The even spreading of AT-MSC-Tk cells within the tumor after 2 weeks of PBS treatment (Fig 4F) is evidence of these cells mobility and interaction with tumor cells. The finding that the AT-MSC-Tk cells remained only within the tumor and its vicinity after GCV treatment (Fig 4C), and not in other brain regions, strengthens this argument. These data and this reasoning support the use of suicide gene therapies, because the fact that suicide gene carriers interact with tumor cells indicates their potentials for achieving better therapeutic efficacy and safety.

An important limitation of this study is the animal GBM model established using U-87 tumor cell line. Although rodent GBM models have been used for decades, the extent to which they reproduce the characteristics found in human GBM remain controversial [50]. Main reasons of using glioma cell lines in rodents are easy to culture for expansion and maintenance, efficient and reproducible to gliomagenesis and easy to locate the injected tumor. However, the degree of tumor necrosis, angiogenesis, endothelial proliferation, invasion and inflammation is significantly variable in comparison to human GBM. Today there are other animal GBM models using different GBM cells, but each model has specific limitations and none of them meet fully human GBM [51].

In conclusion, our data demonstrate that MSCs from adipose tissue are good carriers of the suicide gene HSV-Tk for the treatment of U-87 derived GBM. AT-MSCs are easy to obtain and can be isolated mechanically without exogenous contaminants, making them a strong candidate vehicle for gene therapy of GBM. Gene therapy of this kind, in combination with conventional radiochemotherapy, deserves further evaluation.
